# Prevalence and persistent prescription of analgesic drugs in persons admitted with dementia to a nursing home – A longitudinal study

**DOI:** 10.1371/journal.pone.0279909

**Published:** 2022-12-30

**Authors:** Anne-Sofie Helvik, Sverre Bergh, Kamile Kabukcuoğlu, Jūratė Šaltytė Benth, Bjørn Lichtwarck, Bettina Husebø, Kjerstin Tevik

**Affiliations:** 1 Department of Public Health and Nursing, Faculty of Medicine and Health Sciences, Norwegian University of Science and Technology (NTNU), Trondheim, Norway; 2 Norwegian National Centre for Ageing and Health, Vestfold Hospital Trust, Tønsberg, Norway; 3 Research Centre for Age-related Functional Decline and Disease, Innlandet Hospital Trust, Ottestad, Norway; 4 Faculty of Nursing, Akdeniz University, Campus Antalya, Antalya, Türkiye; 5 Health Services Research Unit, Akershus University Hospital, Lørenskog, Norway; 6 Institute for Clinical Medicine, University of Oslo, Oslo, Norway; 7 Department of Global Public Health and Primary Care, Centre for Elderly and Nursing Home Medicine, University of Bergen, Bergen, Norway; 8 Municipality of Bergen, Bergen, Norway; Xiamen University - Malaysia Campus: Xiamen University - Malaysia, MALAYSIA

## Abstract

The overall aim was to explore the prevalence and persistent regular prescription of opioids and paracetamol among nursing home (NH) residents with dementia at admission and over time. A total of 996 residents with dementia, mean (SD) age 84.5 (7.6) years and (36.1% men), were included at admission (A_1_). Yearly assessments were performed for two years (A_2_ and A_3_) or until death. Pain was assessed using the Mobilization-Observation-Behavior-Intensity-Dementia-2 (MOBID-2) Pain Scale. Information regarding prescription of analgesics, general physical health, personal activities of daily living, severity of dementia, neuropsychiatric symptoms, and prescription of psychotropic drugs was collected. A generalized linear mixed model was used to explore whether pain severity was associated with persistent and persistent prescription of opioids and/or paracetamol across timepoints. At A_1_, 495 of 996 (49.7%) NH residents were prescribed analgesics and prevalence increased at the follow-ups (A_2_: n = 630, 65.1%; A_3_: n = 382, 71.2%). Paracetamol was the most frequently prescribed analgesic at all assessments (A_1_: 45.5%; A_2_: 59.5%; A_3_: 67.1%). Opioid prescriptions were quite prevalent (A_1_: 18.1%; A_2_: 25.1%; A_3_: 28.3%), with odds approximately 13 times (OR = 13.3, 95% CI 6.8–26.0) and 9 times (OR = 8.6, 95% CI 3.7–20.3) higher for prescription at follow-up A_2_ and A_3_, respectively, relative to prescription at A_1_. In adjusted analyses, higher pain intensity and poor physical health were associated with prescription and persistent prescription of opioids and paracetamol. In conclusion, prevalence and persistent prescription of analgesics were high in NH residents with dementia. The odds for the prescription of opioids at follow-up were high if prescribed at baseline. Interdisciplinary collaboration, routine assessment of pain at admission and regularly thereafter, and systematic drug reviews are essential to adequately assess and treat pain in NH residents with dementia.

## Introduction

In Europe and the United States, the majority of people with dementia are in a nursing home (NH) at time of death [1, 2], and studies have reported that up to 85% of NH residents have dementia [3–6]. It is further reported that the prevalence and severity of dementia in NH residents have increased over the years, at least in Norway [3, 4], where the jurisdiction to provide NH care lies with local municipalities [7]. To serve the needs of the country’s 5.4 million inhabitants, there are about 40,000 NH places (beds) [8, 9]. The goals of NHs are to limit negative health consequences of diseases and poor functioning, to promote quality of life (QoL) for older adults, and to provide care and treatment around the clock.

Pain is not only an unpleasant experience but also found to be quite prevalent in NH residents with dementia. A prevalence up to 80% has been reported in these NH residents but it is reported to vary considerably [10–20], which may be due to differences in participant characteristics, pain treatment, definition of pain, and methodology used in the studies [15].

Pain may have negative health consequences, including poorer physical functioning [21–23], depression [14], anxiety [14], agitation [24], and aggression [14], but is also found to limit social interaction [21], and contribute to poor QoL [18, 25, 26]. Pain with an intensity that is considered to affect a person’s function and everyday life is defined as clinically relevant [13] and requires attention and treatment by NH care personnel.

There has been increasing attention directed toward pain in older adults and in NH residents since the first guidelines for the clinical management of chronic pain among older adults were published by the American Geriatric Society (AGS) in 1998 [27]. The International Association for the Study of Pain (IASP) in 2007 implementation of projects to reduce pain in older persons by initiating an international year against pain in older adults [28]. Paracetamol is recommended as the first-line analgesic in older adults, whereas prescription of opioids has been recommended for treatment of moderate to severe pain [29]. Preceding the initiation of pain treatment, a systematic and reliable pain assessment is recommended [27, 30]. During pain treatment, the effect and possible side effects must be considered carefully. Anticholinergic side effects may provoke considerable adverse events in people with dementia [31, 32].

The prevalence of prescribed analgesics in NHs has varied from 35% to 79%, with the highest prevalence in the most recently assessed cohorts [33–38]. The prescription of paracetamol and opioids has especially increased [33–35]. To the best of our knowledge, studies exploring clinically relevant pain and the prescription of analgesics at NH admission in residents with dementia are lacking. However, two cross-sectional studies from 2017 and 2019 including NH residents with dementia independent of length of stay prior to the assessment reported the prevalence of the prescription of any analgesic to be 58% [38] and 61.3% [36] in those with clinically relevant pain, respectively. Those with clinically relevant pain were prescribed analgesics more frequently than those without clinically relevant pain [38]. Paracetamol was most frequently prescribed in both groups [36, 38], but opioids were also prescribed among residents without clinically relevant pain [36, 38].

An association between the degree of pain severity and the prescription of analgesics in NH residents has, as expected, been reported in international epidemiological studies [36, 37]. In addition, other factors indicating poor physical health [36, 39], poorer personal activities of daily living (P-ADL) function [37], affective symptoms [36], and being younger [37, 39] have, in some epidemiological cross-sectional studies, been found to be positively associated with prescription of analgesics in NH residents. Furthermore, some of the epidemiological NH studies have found that those with less-severe cognitive limitation or without dementia were more likely to be prescribed analgesics [39–41], but these results are divergent [17, 37]. It has also been reported that female NH residents with dementia were prescribed analgesics more often [42] than male NH residents with dementia, and that the type of care facility [39] may be associated with prescription of analgesics in these NH residents. However, the results are inconsistent with other studies’ findings in regard to prescription among female residents with dementia [36, 39] and with respect to care facility [36].

To the best of our knowledge, there are few epidemiological longitudinal studies that have assessed prescription of analgesics in NH residents with dementia from admission, with the exception of a relatively small study from the Netherlands (information about analgesics from n = 171 residents at baseline) [10]. However, that study did not assess clinically relevant pain, investigate if or how degree of pain was associated with prescribing opioids and paracetamol, or examine factors associated with persistent prescription of analgesics over time [10].

In the present study, the first aim was to assess the prevalence of analgesic drug prescription among NH residents with dementia at admission and after 12 and 24 months and persistent analgesic drug prescription from admission to 12 months and from 12 to 24 months in the entire sample, as well as stratified by clinically relevant pain. The second aim was to assess prescription of analgesic drugs in relation to prescription one or two timepoints earlier (persistent prescription) and to explore factors associated with persistent analgesic drug prescription. The third aim was to explore whether there is an effect of pain on prescription of these analgesics over time.

## Materials and methods

### Design

This was an observational, longitudinal study of newly admitted NH residents from a convenience sample of 68 NHs in South-Eastern Norway where baseline data were collected from November 2014 to December 2019. The NHs were located in 32 municipalities representing NHs in rural and urban areas of one county and were all non-profit NHs operated and owned by the municipalities. The follow-up data were collected annually or until the participant’s death. Follow-up assessments are ongoing, but the present study includes information from baseline (A_1_) to two years (A_3_), with data collection completed by the end of 2021.

### Participation and setting

During 2014–2019, a total of 3,318 residents were registered as admitted to one of the 68 participating NHs. For the present study, 1,283 residents with an expected stay longer than four weeks were recruited. All residents 65 years and older, independent of whether they had established dementia or not, and residents younger than 65 with established dementia were asked to participate. The only exclusion criterion was life expectancy less than six weeks. The persons not recruited (2,035), due either to death shortly after admission (238), not accepting the invitation (567), or other unknown reasons (1,230), were more often women, but they did not differ in age from those recruited, i.e., mean (SD) age 84.2 (862) and 84.53 (7.8) years, respectively.

**Fig 1:**
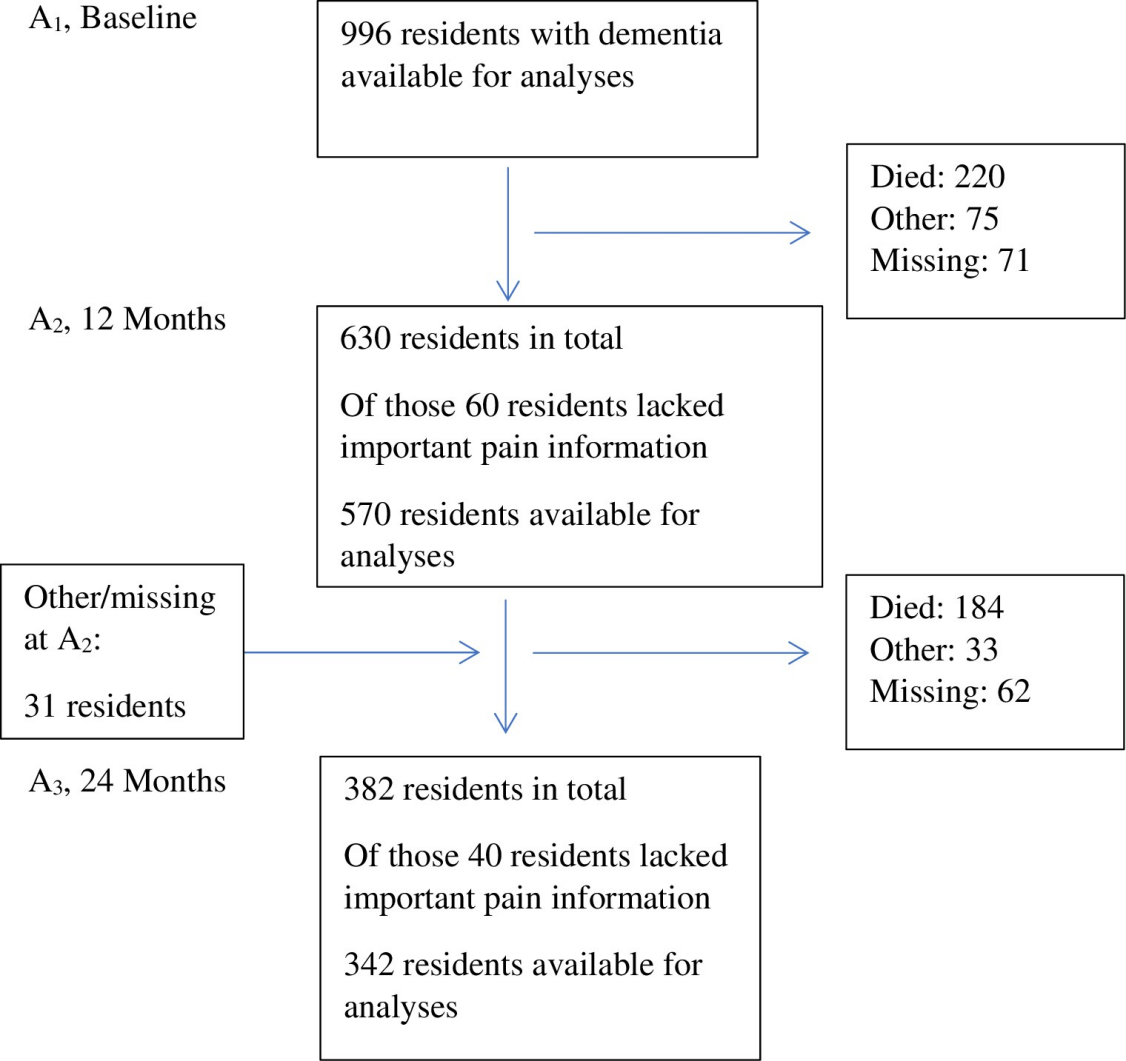
Flowchart.

The present study included only residents with dementia at admission. Based on all available information, two physicians independently diagnosed dementia at admission according to the ICD-10 criteria [43]; a third was consulted in situations where the two physicians disagreed. All physicians had extensive experience with research and clinical old-age psychiatry. In total, 1,074 residents had dementia, 201 did not have dementia, and 8 could not be diagnosed. Of those with dementia, 78 residents lacked information about pain severity. Thus, the present study at baseline (A_1_) included 996 residents with dementia admitted to a NH. At the follow-ups, the numbers of residents available for analyses were 570 at A_2_ and 342 at A_3_ (Fig 1). Mean (SD) duration from A_1_–A_2_ and from A_2_–A_3_ was 341 (82.7) and 356 (66.9) days, respectively. Mean (SD) time of follow-up was 683 (98.2) days.

### Measures

The Mobilization-Observation-Behavior-Intensity-Dementia-2 (MOBID-2) Pain Scale is an observational tool for people with dementia and was used at all assessments [44, 45]. MOBID-2 assesses nociceptive, musculoskeletal pain during active, guided movements and pain that might be related to internal organs, the head, and the skin during the previous week, documented on a body chart to show potential pain location. The pain scale comprises 10 single items scored from 0 (no pain) to 10 (most severe pain), with a maximum sum score of 100.

An additional item evaluates overall pain intensity from 0 to 10 (most-severe pain intensity). An overall pain-intensity score of ≥3 indicates that the resident has clinically relevant pain intensity [44]. Nursing staff who knew each resident best completed the scale. The MOBID-2 Pain Scale’s validity, reliability, and responsiveness have been assessed, and it has been used in several studies of NH residents including in Norway [45–47].

The prescription of regular analgesics was documented from each resident’s medical record at all assessments. Medications were grouped according to the Anatomical Therapeutic Chemical (ATC) classification system [48]. ATC codes beginning with N02 were divided into opioids (N02A) and paracetamol (N02B E01, N02A J06 & N02A J13) [48]. The prescription of each of these analgesics as well as “any type” was dichotomized as yes or no.

Psychotropic drugs were categorized as antipsychotics (N05A except lithium), antidepressants (N06A), anxiolytics (N05B), and hypnotics/sedatives (N05C) [48]. Prescription of each type of these psychotropic drugs as well as “any type” was dichotomized as yes or no. Additionally, the prescription of any type of psychotropic drug was summarized as the prescription of 0, 1, 2 or ≥3 drugs.

The Neuropsychiatric Inventory–Nursing Home version (NPI-NH) was used at all assessments to measure neuropsychiatric symptoms (NPS) [49]. The 12-item inventory assesses the following symptoms: delusion, hallucination, euphoria, agitation/aggression, disinhibition, irritability/lability, depression/dysphoria, anxiety, apathy/indifference, aberrant motor behavior, night-time behavior disturbances, and appetite and eating disorders (yes/no). For each symptom present, severity (score 1–3) and frequency (score 1–4) are measured. Frequency and severity scores are then multiplied, resulting in a score 0–12 for each symptom. Three sub-syndromes of NPS have been established by factor analysis: psychosis (including delusions and hallucinations), agitation (agitation/aggression, disinhibition, and irritability), and affective (depression and anxiety) [50]. The NPI-NH was translated to Norwegian and validated in 2008 [51].

The severity of dementia was measured using the Clinical Dementia Rating (CDR) scale at each assessment, which assesses six domains: memory, orientation, judgment and problem-solving, community affairs, home and hobbies, and personal care [52]. A total score with five response categories (0, 0.5, 1, 2, and 3) is calculated using an algorithm that prioritizes memory [52, 53]. The categories indicate level of dementia ranging from 0 (no dementia) to 3 (severe dementia). A sum score of the six domains (CDR Sum of Boxes, CDR-SoB) ranging from 0 to 18 offers important advantages when analyzing data. A higher score indicates more-severe dementia. The correlation between the categorical CDR and the CDR-SoB is high [54, 55]. The CDR scale has been translated to Norwegian and used in several NH studies [4, 46].

The Physical Self-Maintenance Scale (PSMS) [56] was used at all assessments to assess P-ADL. The PSMS includes six items with a total score ranging from 6 (highest possible level of functioning) to 30 (poorest possible level of functioning) [56]. The nursing personnel knowing the resident best completed the scale, which is used frequently in Norwegian NH studies [57, 58].

The General Medical Health Rating (GMHR), used at all assessments to measure physical health, is a one-item global rating scale with four response alternatives: excellent, good, fair, and poor [59]. The rating was based on all available information of physical health and use of prescribed medication. The scale has previously been used in large NH studies, including in Norway with NH residents with dementia [57]. For analyses, the scale was dichotomized to either poor (including fair and poor) or good (excellent and good) physical health [18].

Demographic information (age, sex, and marital status) was collected from medical records at baseline. Marital status (married or partner) was recorded at all assessments. The type of NH unit was reported at baseline and categorized either as a regular unit (RU) or a special care unit for people with dementia (SCU).

### Procedure

Data were collected by healthcare workers, mainly registered nursing staff (74%), at the NHs and supervised by 10 research nurses. Data collectors completed a two-day training program prior to the data collection. All baseline information regarding each resident was collected over the first month of the NH stay, and data came from a standardized interview with the residents, their next of kin, their caregivers in the NH, and from medical records.

The NH staff, including the NH physician, assessed residents’ capacity to consent to participate in the study, and all residents who had the capacity gave their written consent. If a resident’s capacity to consent was reduced, his or her next of kin consented on the resident’s behalf. These procedures have been recommended and approved by the Norwegian Regional Ethics Committee South-East (2014/917).

### Statistics

Baseline characteristics were presented as means and standard deviations (SDs) or frequencies and percentages, as appropriate. As data were collected at different NHs, a hierarchical structure is likely present. Residents prescribed and not prescribed analgesics were, therefore, compared by generalized linear mixed model with random intercepts for NHs.

Prevalence and persistent prescription (prescribed at two consecutive timepoints) of analgesics were presented as percentages for the entire sample as well as stratified by dichotomized overall pain-intensity score (MOBID-2 <3 vs. MOBID-2 ≥3) as assessed at baseline. The groups were compared by generalized linear mixed model with random intercepts for NHs.

The odds for the prescription of analgesic drugs (opioids or paracetamol) at one timepoint, adjusted for prescription of the same drug at a previous timepoint (Lag 1) or two timepoints earlier (Lag 2), were assessed by bivariate generalized linear mixed model with random intercepts for residents nested within NHs. In this model, the outcome was the prescription of an analgesic drug at A_2_ (12 months) or A_3_ (24 months), while the main factor was the prescription of the same drug at baseline (A_1_). The models were further adjusted for preselected covariates (age, sex, marital/partner status, MOBID-2 sum score, CDR-SoB, dichotomized GMHR, PSMS score, NPI-NH agitation sub-syndrome score, NPI-NH affective sub-syndrome score, NPI-NH psychosis sub-syndrome score, NPI-NH apathy score, prescription of psychotropic drugs (0, 1, 2, ≥ 3), and type of NH unit) measured at baseline.

Factors associated with persistent prescription of analgesic drugs at two consecutive timepoints were assessed using a generalized linear mixed model with random intercepts for residents nested within NHs. The model included fixed effects for factors assessed at the earlier of the two consecutive timepoints and was adjusted for the same covariates as above measured simultaneously with outcome whenever possible.

To assess whether pain intensity measured by the MOBID-2 sum score was associated with trend in the odds for prescribing analgesic drugs, a generalized linear mixed model with random effects for patients nested within NH was estimated. The model included fixed effects for time (coded as dummies), MOBID-2 sum score assessed simultaneously with prescription of analgesics, and the interaction between the two. A significant interaction would imply that the association between the prescription of analgesics and MOBID-2 sum score varied with time. The model was adjusted for preselected covariates (reported above) measured simultaneously with outcome whenever possible.

Only cases with no missing values for covariates were included in the regression analyses. All tests were two-sided, and results with p-values below 0.05 were considered significant. The statistical analyses were performed in SPSS version 27 and STATA version 17.

## Results

Mean (SD) age was 84.5 (7.6) years, and 360 (36.1%) were men. Mean (SD) CDR-SoB was 11.2 (3.5). NH residents with dementia who were prescribed analgesics (paracetamol and/or opioids) were older, more often in poor physical health, had higher mean PSMS scores (poorer P-ADL functioning), and higher mean agitation sub-syndrome NPI-NH scores compared to those not prescribed analgesics (Table 1). Additionally, those prescribed analgesics were more often prescribed psychotropic drugs and were in regular care units than those not prescribed analgesics.

**Table 1:** Sample characteristics of newly admitted NH residents with dementia (N = 996).

Characteristics	Analgesics n = 495 (49.7%)	No analgesics n = 501 (50.3%)	p-value ^1^
*Socio-demographics*			
Age, mean (SD)	85.4 (7.3)	83.7 (7.8)	< 0.001
Males, n (%)	144 (29.1)	216 (43.1)	< 0.001
Married/Cohabitant, n (%) (11 missing)	138 (28.3)	172 (34.5)	0.065
*Health condition*			
CDR-SoB, mean (SD) (41 missing)	11.3 (3.6)	11.2 (3.4)	0.451
GMHR, n (%) (65 missing)			
Fairly poor/Poor	277 (59.8)	203 (43.4)	< 0.001
Good/Fairly good	186 (40.2)	265 (56.6)	
PSMS score, mean (SD) (4 missing)	15.8 (4.5)	14.2 (4.3)	< 0.001
NPI-NH sub-syndrome^2^			
Agitation, mean (SD) (30 missing)	5.5 (8.1)	4.4 (7.0)	0.009
Affective, mean (SD) (33 missing)	3.7 (5.3)	3.4 (5.3)	0.338
Psychosis, mean (SD) (21 missing)	2.1 (4.3)	1.9 (3.8)	0.272
Apathy, mean (SD) (21 missing)	1.2 (2.6)	0.9 (2.22)	0.093
Use of psychotropic medication (yes), n (%)			
Antipsychotics	58 (11.7)	56 (11.2)	0.759
Antidepressants	165 (33.3)	137 (27.4)	0.058
Anxiolytics	73 (14.8)	67 (13.4)	0.757
Sedatives	141 (28.5)	110 (22.0)	0.027
Any	296 (59.8)	253 (50.5)	0.005
MOBID-2 sum score ^3^, mean (SD)	10.9 (10.9)	5.3 (6.5)	< 0.001
*NH characteristics*, n (%) (18 missing)			
Regular care unit	310 (63.8)	254 (51.6)	< 0.001
Special care unit	176 (36.2)	238 (48.4)	
*Type of dementia*, n (%)			
Alzheimer’s disease	306 (61.8)	309 (61.7)	0.949
Vascular dementia	35 (7.1)	32 (6.4)	
Alzheimer’s disease mixed type	51 (10.3)	57 (11.4)	
Frontotemporal dementia	46 (9.3)	48 (9.6)	
Lewy body dementia/ Parkinson’s disease	40 (8.1)	46 (9.2)	
Unspecified	17 (3.4)	9 (1.8)	

Analgesics includes either Opioids or Paracetamol or both

Abbreviations: CDR-SoB = Clinical Dementia Rating—Sum of Boxes; GMHR = General Medical Health Rating; MOBID-2 = Mobilization-Observation-Behavior-Intensity-Dementia-2; n = number; NH = Nursing Home; NPI-NH = Neuropsychiatric Inventory Nursing Home version; PSMS = Physical Self-Maintenance Scale; SD = Standard Deviation.

^1^Generalized linear mixed model (adjusting for cluster effect within NH)

^2^ NPI-NH Agitation sub-syndrome: agitation/aggression, disinhibition and irritability, NPI-NH Affective sub-syndrome: depression and anxiety, NPI-NH sub-syndrome psychosis: delusions and hallucination.

^3^The sum score from the ten single MOBID-2 items

### Prevalence and persistent prescription of analgesics

Of 996 participants, 495 (49.7%) were prescribed paracetamol and/or opioids at baseline (Table 2). The prevalence of any of the analgesics being prescribed increased at the follow-ups (A_2_ (n = 630, 65.1%) and A_3_ (n = 382, 71.2%)). Paracetamol was prescribed most frequently at all assessments (A_1_: 45.5%; A_2_: 59.5%; A_3_: 67.1%).

**Table 2:** Prevalence and persistent use of analgesic drugs.

	Prevalence					Persistent use				
	A1		A2		A3		A1-A2		A2-A3	
	All (N = 996)	MOBID-2≥3^§^ Yes/No (N = 355/641)	All (N = 630)	MOBID-2≥3^§^ Yes/No (N = 206/424)	All (N = 382)	MOBID-2≥3^§^ Yes/No (N = 122/260)	All (N = 570)	MOBID-2≥3^§^ Yes/No (N = 187/383)	All (N = 318)	MOBID-2≥3^§^ Yes/No (N = 102/216)
Opioids	18.1	30.1/11.4*	25.1	44.7/15.6*	28.3	41.0/22.3*	11.2	22.5/5.7*	17.9	29.4/12.5*
Paracetamol	45.5	60.0/37.4*	59.5	71.4/53.8*	67.0	80.3/60.8*	36.3	50.3/29.5*	52.2	69.6/44.0*
Any analgesic drug	49.7	66.8/40.2*	65.1	80.1/57.8**	71.2	82.8/65.8**	41.8	60.4/32.6*	56.0	72.6/48.2*

Note: A1-A3: Assessment 1 = baseline; Assessment 2 = 12 months; Assessment 3 = 24 months; Any analgesic drugs: use of Opioids and/or Paracetamol

Abbreviation: MOBID-2 = Mobilization-Observation-Behavior-Intensity-Dementia-2.

^§^ MOBID-2 ≥3 at **baseline was used.**

*p < 0.001 for generalized linear mixed model (adjusting for cluster effect within NH);

**p < 0.01 for generalized linear mixed model (adjusting for cluster effect within NH)

The prevalence of clinically relevant pain (MOBID-2 ≥3) at each timepoint varied, i.e., 35.6% (N = 355/996) at A_1_, 37.7% (N = 215/570) at A_2_, and 41.5% (N = 142/342) at A_3_. At all assessments, the prevalence of any analgesics was higher for those with clinically relevant pain (MOBID-2 ≥3) than for those without clinically relevant pain at baseline.

The persistent prescription of any analgesics between two consecutive assessments was high (>40%) throughout the study period. The persistent prescription of paracetamol between two assessments was higher than the proportion of persistent prescription of opioids between the same assessments. The proportion of participants with persistent prescription of any analgesics between two consecutive assessments was higher in those with clinically relevant pain (MOBID-2 ≥3) at baseline than without clinically relevant pain.

Unadjusted and adjusted odds for the prescription of opioids and paracetamol at one timepoint, given prescription of the same type of analgesics at an earlier timepoint, were high (Table 3). The odds for the persistent prescription of analgesics were highest when compared with prescription at the nearest assessment (Lag 1) compared to odds for prescription two timepoints apart (Lag 2). All results were highly significant (p<0.001).

**Table 3:** Analgesic drug use at one time adjusted for use one or two time points earlier ^1^. Results of generalized linear mixed model.

	Unadjusted models		Adjusted model^2^	
	OR (95% CI)	p-value	OR (95% CI)	p-value
	*Lag 1 (N = 526)*			
Opioids	14.0 (7.9; 24.7)	< 0.001	13.3 (6.8; 26.0)	< 0.001
Paracetamol	8.0 (5.1; 12.5)	< 0.001	7.5 (4.6; 12.2)	< 0.001
	***Lag 2 (N = 320)***			
Opioids	7.7 (3.8; 15.6)	< 0.001	8.6 (3.7; 20.3)	< 0.001
Paracetamol	3.7 (2.1; 6.3)	< 0.001	3.3 (1.8; 5.9)	< 0.001

Note: Lag 1: One time point earlier (use at A2 adjusted for use at baseline). Lag 2: Two time points apart

(use at A3 adjusted for use at baseline).

^1^ Use of analgesics at baseline is main factor in all models; All analyses adjusted for cluster effect within NH; Only cases with no missing values on adjustment variables are included in the analyses.

^2^ Adjusted for age, gender, marital status, MOBID-2 sum score, CDR-SoB, dichotomized GMHR, PSMS score, NPI-NH agitation sub-syndrome score, NPI-NH affective sub-syndrome score, NPI-NH psychosis sub-syndrome score, NPI-NH apathy score, use of psychotropic drugs (0, 1, 2, ≥ 3) and type of NH-unit at baseline.

Abbreviations: CDR-SoB = Clinical Dementia Rating—Sum of Boxes; CI = Confidence Interval; GMHR = General Medical Health Rating; MOBID-2 = Mobilization-Observation-Behavior-Intensity-Dementia-2; NH = Nursing Home; NPI-NH = Neuropsychiatric Inventory Nursing Home version; OR = Odds Ratio; PSMS = Physical Self-Maintenance Scale.

NPI-NH Agitation sub-syndrome: agitation/aggression, disinhibition and irritability, NPI-NH Affective sub-syndrome: depression and anxiety, NPI-NH sub-syndrome psychosis: delusions and hallucination,

### Factors associated with prescription of analgesics at two consecutive timepoints

The adjusted odds for the persistent prescription of opioids at two consecutive timepoints were elevated among residents with higher pain-intensity scores (MOBID-2 sum score), poor physical health (GMHR), lower apathy symptom score, having been prescribed one or three or more psychotropic drugs (compared to none), or having been married/partner at the first of the two assessments (Table 4). The adjusted odds for the persistent prescription of paracetamol were elevated among residents with higher pain-intensity scores (MOBID-2 sum score), poor physical health (GMHR), or having been prescribed one psychotropic drug medication regularly (compared to none) at the first of the two assessments.

**Table 4:** Factors associated with persistent prescription of analgesics at two consecutive time points. Results of generalized linear mixed model^1^ (N = 818).

	Opioids				Paracetamol			
	Unadjusted models		Adjusted model		Unadjusted models		Adjusted model	
	OR (95% CI)	p-value	OR (95% CI)	p-value	OR (95% CI)	p-value	OR (95% CI)	p-value
*Factors assessed at the first two consecutive time points*								
MOBID-2	1.09 (1.07; 1.11)	**<0.001**	1.11 (1.07; 1.14)	**<0.001**	1.09 (1.05; 1.12)	**<0.001**	1.07 (1.03; 1.11)	**0.001**
CDR-SoB	1.04 (0.97; 1.10)	0.267	0.93 (0.83; 1.05)	0.255	1.11 (1.02; 1.21)	**0.018**	0.99 (0.90; 1.10)	0.876
GMHR								
Poor–ref.	1		1		1		1	
Good	0.38 (0.24; 0.59)	**<0.001**	0.42 (0.23; 0.79)	**0.006**	0.36 (0.21; 0.62)	**<0.001**	0.54 (0.30; 0.95)	**0.032**
PSMS	1.09 (1.04; 1.15)	**<0.001**	1.06 (0.97; 1.15)	0.188	1.18 (1.10; 1.28)	**<0.001**	1.07 (0.98; 1.16)	0.129
NPI-NH sub-syndrome ^2^								
Agitation	1.02 (0.99; 1.05)	0.102	1.01 (0.97; 1.06)	0.581	1.02 (0.98; 1.05)	0.315	1.02 (0.98; 1.06)	0.442
Affective	1.04 (1.00; 1.09)	**0.031**	1.03 (0.97; 1.10)	0.359	1.00 (0.95; 1.06)	0.871	0.98 (0.91; 1.04)	0.481
Psychosis	1.02 (0.97; 1.07)	0.460	0.98 (0.91; 1.06)	0.598	1.02 (0.95; 1.08)	0.640	0.97 (0.90; 1.04)	0.386
Apathy	0.89 (0.78; 1.00)	0.059	0.83 (0.70; 0.99)	**0.041**	1.01 (0.91; 1.13)	0.831	1.02 (0.91; 1.14)	0.710
Use of PTD								
0 –ref.	1		1		1		1	
1	1.28 (0.77; 2.13)	0.346	2.60 (1.24; 5.44)	**0.011**	3.16 (1.56; 6.43)	**0.001**	2.39 (1.15; 4.95)	**0.019**
2	1.75 (0.98; 3.13)	0.057	2.06 (0.86; 4.93)	0.105	3.62 (1.55; 8.45)	**0.003**	1.67 (0.75; 3.71)	0.212
3+	1.97 (0.92; 4.21)	0.083	3.02 (1.04; 8.76)	**0.041**	4.03 (1.26; 12.89)	**0.019**	3.30 (0.98; 11.08)	0.053
Civil status								
Unmarried/no partner–ref.	1		1		1		1	
Married/partner	1.13 (0.71; 1.78)	0.613	2.06 (1.03; 4.13)	**0.041**	0.82 (0.43; 1.56)	0.541	1.33 (0.71; 2.47)	0.372
*Factors assessed at baseline*								
Age	1.03 (0.99; 1.06)	0.066	1.04 (0.99; 1.08)	0.120	1.04 (0.99; 1.08)	0.057	1.04 (0.99; 1.08)	0.084
Gender								
Females–ref.	1		1		1		1	
Males	0.58 (0.36; 0.92)	**0.023**	0.66 (0.33; 1.29)	0.223	0.57 (0.29; 1.05)	0.072	0.58 (0.31; 1.08)	0.084
NH care unit								
Regular–ref.	1		1		1		1	
Special	0.58 (0.33; 1.02)	0.059	0.88 (0.42; 1.83)	0.731	0.60 (0.37; 0.97)	**0.036**	0.87 (0.48; 1.55)	0.627

Note: Bold values shown statistically significant result with a p-value less than 0.05.

Abbreviations: CI = confidence interval; CDR-SoB = Clinical Dementia Rating–Sum of Boxes; GMHR = General Medical Health Rating; MOBID-2 = Mobilization-Observation-Behavior-Intensity-Dementia-2; NH = Nursing Home; NPI-NH, Neuropsychiatric Inventory Nursing Home version; OR = Odds Ratio; PSMS = Physical Self- Maintenance Scale; PTD = Psychotropic drugs

^1^ All analyses adjusted for cluster effect within NH; Only cases with no missing values on adjustment variables are included in the analyses,

^2^ NPI-NH Agitation sub-syndrome: agitation/aggression, disinhibition and irritability, NPI-NH Affective sub-syndrome: depression and anxiety, NPI-NH sub-syndrome psychosis: delusions and hallucination.

### Pain intensity and its association with prescription of analgesics at the same timepoint

In an adjusted analysis of the prescription of opioids and paracetamol, interaction between time and pain-intensity score (MOBID-2 sum score) was not significant, implying that the association between prescription and pain intensity did not vary with time. Overall, a higher pain-intensity score was significantly associated with higher odds for prescription of these analgesics assessed simultaneously (Table 5).

**Table 5:** Factors associated with prescription of analgesics. Results of generalized linear mixed model^1^ (N = 1645).

	Opioids			Paracetamol				
	Unadjusted models		Adjusted model	Unadjusted models			Adjusted model	
	RC (SE)*	p-value	RC (SE)*	p-value	RC (SE)*	p-value	RC (SE)*	p-value
Time								
0	0		0		0		0	
12	0.47 (0.21)	**0.029**	0.38 (0.38)	0.319	1.20 (0.24)	**<0.001**	0.69 (0.40)	0.083
24	0.60 (0.25)	**0.017**	0.47 (0.47)	0.318	1.79 (0.31)	**<0.001**	0.98 (0.51)	0.055
MOBID-2	0.08 (0.01)	**<0.001**	0.07 (0.01)	**<0.001**	0.10 (0.01)	**<0.001**	0.07 (0.01)	**<0.001**
Time x MOBID-2								
0	0		0		0		0	
12	-0.02 (0.01)	0.221	-0.001 (0.02)	0.953	-0.02 (0.02)	0.300	0.05 (0.04)	0.228
24	-0.02 (0.02)	0.332	0.006 (0.03)	0.819	-0.03 (0.02)	0.253	-0.04 (0.03)	0.290
	OR (95% CI)	p-value	OR (95% CI)	p-value	OR (95% CI)	p-value	OR (95% CI)	p-value
*Covariates assessed simultaneously with outcome*								
CDR-SoB	1.03 (0.99; 1.07)	0.172	0.97 (0.91; 1.03)	0.334	1.04 (0.99; 1.10)	0.118	0.97 (0.90; 1.04)	0.380
GMHR								
Poor–ref.	1		1		1		1	
Good	0.48 (0.36; 0.63)	**<0.001**	0.49 (0.34; 0.72)	**<0.001**	0.45 (0.32; 0.64)	**<0.001**	0.56 (0.38; 0.84)	**0.005**
PSMS	1.05 (1.02; 1.08)	**0.002**	1.04 (0.99; 1.09)	0.099	1.09 (1.05; 1.14)	**<0.001**	1.08 (1.02; 1.14)	**0.005**
NPI-NH sub-syndrome^2^								
Agitation	1.01 (0.99; 1.03)	0.324	1.01 (0.98; 1.03)	0.577	1.01 (0.99; 1.03)	0.287	1.02 (0.99; 1.05)	0.139
Affective	1.01 (0.98; 1.03)	0.618	0.99 (0.95; 1.03)	0.678	0.99 (0.96; 1.03)	0.702	0.97 (0.93; 1.01)	0.173
Psychosis	1.00 (0.97; 1.03)	0.999	0.98 (0.94; 1.03)	0.487	1.00 (0.96; 1.04)	0.897	0.99 (0.94; 1.05)	0.763
Apathy	0.95 (0.89; 1.01)	0.076	0.93 (0.86; 1.00)	0.062	1.01 (0.94; 1.08)	0.811	1.01 (0.94; 1.10)	0.711
Use of PTD								
0 –ref.	1		1		1		1	
1	1.14 (0.83; 1.56)	0.423	1.46 (0.97; 2.20)	0.070	1.85 (1.23; 2.79)	**0.003**	1.62 (1.04; 2.52)	**0.033**
2	1.28 (0.89; 1.85)	0.180	1.16 (0.70; 1.93)	0.562	1.86 (1.14; 3.05)	**0.013**	1.38 (0.80; 2.41)	0.250
3+	1.40 (0.89; 2.20)	0.144	1.20 (0.63; 2.28)	0.587	2.06 (1.06; 3.99)	**0.033**	1.61 (0.77; 3.39)	0.208
Civil status								
Unmarried/no partner–ref.	1		1		1		1	
Married/partner	1.08 (0.81; 1.44)	0.599	1.16 (0.78; 1.74)	0.465	0.70 (0.46; 1.07)	0.098	0.89 (0.58; 1.38)	0.615
*Covariates assessed at baseline*								
Age	1.02 (1.00; 1.04)	**0.046**	1.02 (0.99; 1.05)	0.139	1.04 (1.01; 1.07)	**0.004**	1.03 (1.00; 1.06)	**0.029**
Gender								
Females–ref.	1		1		1		1	
Males	0.72 (0.54; 0.96)	**0.025**	0.76 (0.51; 1.12)	0.160	0.57 (0.38; 0.87)	**0.009**	0.48 (0.31; 0.75)	**0.001**
NH care unit								
Regular–ref.	1		1		1		1	
Special	0.71 (0.50; 1.01)	0.060	0.86 (0.58; 1.27)	0.449	0.60 (0.41; 0.87)	**0.007**	0.76 (0.51; 1.14)	0.184

Note: Bold values shown statistically significant result with a p-value less than 0.05.

Abbreviations: CDR-SoB = Clinical Dementia Rating–Sum of Boxes; GMHR = General Medical Health Rating; MOBID-2 = Mobilization-Observation-Behavior-Intensity-Dementia-2; NH = Nursing Home; NPI-NH, Neuropsychiatric Inventory Nursing Home version; PSMS = Physical Self- Maintenance Scale; PTD = Psychotropic drugs; RC = Regression Coefficient; SE = Standard Error.

^1^ All analyses adjusted for cluster effect within NH; Only cases with no missing values on adjustment variables are included in the analyses,

^2^ NPI-NH Agitation sub-syndrome: agitation/aggression, disinhibition and irritability, NPI-NH Affective sub-syndrome: depression and anxiety, NPI-NH sub-syndrome psychosis: delusions and hallucination.

*Regression coefficients (RC) and standard errors (SE) are presented instead of odds ratios and confidence intervals due to interaction

In addition, poor physical health was associated with increased odds for prescription of opioids. Furthermore, having poor physical health, low P-ADL functioning (higher PSMS score), or having been prescribed one psychotropic drug (compared to none) increased the odds for the prescription of paracetamol. Older age or being female increased the odds for prescribing paracetamol.

## Discussion

The prevalence and persistence of the prescription of any analgesics were high from admission to NH and throughout the 24-month study period, with prevalence ranging from 50% (A_1_) to 71% (A_3_) and persistence from 42% (A_1_–A_2_) to 56% (A_2_–A_3_). Paracetamol was most frequently prescribed at all three assessments. NH residents with clinically relevant pain assessed at baseline (MOBID-2 ≥3) were more frequently prescribed opioids and paracetamol and more often had a persistent prescription for these drugs throughout the study period than those without clinically relevant pain at baseline. The odds for the prescription of opioids and paracetamol at 12-month follow-up when prescribed the same category of analgesics at baseline were higher than at 24-month follow-up when prescribed the same analgesics at baseline. Higher pain intensity (MOBID-2 sum score) was associated with the persistent prescription of both categories of analgesics, and pain intensity was associated with prescription of both opioids and paracetamol, also when adjusting for factors assessed simultaneously. In addition, having poor health increased the odds for the prescription of analgesics when assessed simultaneously.

The high prevalence of the prescription of analgesics found in the present study at NH admission is comparable with recent cross-sectional studies in Europe and Australia that included NH residents with dementia independent of length of stay prior to assessment [36–38]. Furthermore, NH residents at baseline were most frequently prescribed paracetamol, as found in previous studies assessing NH residents with dementia [10, 36–38]. Throughout the study and independent of degree of pain intensity, paracetamol was the most frequently prescribed analgesic drug. The prevalence of its prescription varied between 46% and 67%, which is in line with recommendations for prescribing paracetamol as the first-line analgesic for older adults [29, 31, 60] and people with dementia [31]. Paracetamol appears safe to prescribe at recommended doses to older adults [31, 61, 62] and people with dementia [31]; however, we have limited knowledge about adverse events related to its long-term use for people with dementia [32] as well as whether it works at all.

Even so, the prescription of opioids among participants was prevalent throughout the study (A_1_: 18.1%; A_2_: 25.1%; A_3_: 28.3%). The prevalence at admission was in line with the prevalence of opioids found in previous cross-sectional studies of NH residents with dementia independent of length of stay prior to assessment, i.e., in previous Norwegian studies (2019: 19.3%; 2016: 17.9%) [34, 36] and studies from other European countries (Finland, 2011: 13.5%; Ireland, 2015: 14.3%) [63, 64].

When assessing prescription of analgesics among NH residents with clinically relevant pain intensity at baseline (MOBID-2 ≥3), we found that 60% of those with clinically relevant pain were prescribed analgesics at NH admission (A_1_), while 40% of those without clinically relevant pain were prescribed analgesics. In total, 11% of the residents without clinically relevant pain were prescribed opioids at A_1_; these findings are in line with two previous cross-sectional studies including NH residents with dementia independent of length of stay when assessed [36, 38]. In the present study, it seems that, at the follow-ups, the proportion of residents with baseline clinically relevant pain being prescribed any analgesics or opioids had increased (80.1% and 44.7% at A_2_ and 82.8% and 41.2% at A_3_, respectively). However, for those with no clinically relevant pain at baseline, the proportion being prescribed any analgesics or opioids had increased, i.e., 58% and 16% at A_2_ and 66% and 22% at A_3_, respectively. The present study does not have information about the effect of treatment. At admission, we do not know whether those being prescribed analgesics and being without clinically relevant pain did not have clinically pain due to a treatment effect of the given analgesics or not, or whether a clinically relevant pain intensity will occur if the analgesics are terminated. However, we suggest that those with clinically relevant pain at baseline, independent of whether they were prescribed analgesics or not, could profit from a throughout evaluation of their pain treatment.

The present study adds to the limited literature by assessing NH residents with dementia at admission and using a longitudinal, observational design focusing on both the prevalence and persistent prescription of opioids and paracetamol. A relatively small (n = 171) longitudinal NH study from the Netherlands reported the prevalence and persistent prescription of paracetamol beginning at admission in residents with dementia [10] and following them with biannual assessments for 3.5 years. Compared with our study, the Netherlands study found a somewhat lower prevalence (34%–52%) and a higher proportion of the persistent (48%–80%) prescription of paracetamol [10]. However, it did not report the prevalence and persistent prescription of opioids [10]. In our study we found that, even if relatively few residents were prescribed opioids and the proportion with persistent opioid prescription was low (11%–18%), the odds for opioids at follow-ups A_2_ and A_3_ were almost 13 times and 9 times higher when these were prescribed at baseline compared to participants without opioids at baseline. Furthermore, a relatively large number of participants were prescribed paracetamol, and the proportion with persistent prescription was prominent (36%–52%), but the odds for persistent prescription were somewhat more moderate although higher when comparing the prescription of paracetamol with the nearest assessment timepoint, i.e., 8 times and 3 times higher. These findings may indicate that, when opioids were prescribed at baseline, NH residents with dementia were more likely to continue taking opioids than when paracetamol was prescribed at baseline. Persistent use of opioids might be a concern for these residents since it may increase the risk of serious adverse events due to anticholinergic side effects, comorbidities, polypharmacy, and changes in pharmacokinetics and pharmacodynamics [31, 32, 61]. Thus, treating residents with dementia with opioids should include a careful assessment of the benefits and the risks for each resident [31] and should be discontinued when possible [65]. Moreover, according to Norwegian guidelines [65], opioid use should be restricted in the treatment of chronic non-malignant pain because of the risk of addiction and serious adverse events.

In adjusted generalized linear mixed model analyses, higher pain intensity (MOBID-2 sum score) was associated with higher odds for persistent prescription of both opioids and paracetamol at two consecutive assessments and being prescribed opioids and paracetamol at the same assessment. These findings are as expected and comparable to cross-sectional studies reporting prescription of opioids according to pain intensity [36]. Additionally, we found that the prescription of psychotropic drugs at one timepoint was associated with increased odds of prescription of opioids and paracetamol when assessed simultaneously and persistent prescription of paracetamol at two consecutive assessments. We do not have a firm explanation for these findings. Even so, typical pain-related behaviors in persons with dementia, such as verbalization/vocalization (e.g., sighing, moaning, calling out, gasping), facial expressions (e.g., grimacing, frowning), and defensive postures (e.g., freezing, tensing, guarding, pushing, crouching) [27, 66–68] may also be related to dementia [67]. Typical pain symptoms may be difficult to interpret [67]; therefore, this group may be prescribed both analgesics and psychotropic drugs. However, these are speculations and require further investigation. It should also be noted that neuropsychiatric sub-syndrome scores were not associated with the prevalence or persistent prescription of analgesics, although cross-sectional studies have found higher affective syndrome scores linked to prescription of analgesics [36].

### Strengths and limitations

A major strength of this study is its methodology, mainly the use of a well-known, internationally recognized scale for assessing pain, MOBID-2 [44, 45]; the use of a measure of cognitive functioning; and the experience of the research institution with such studies [4, 46]. The large sample size allowed us to adjust for several factors known to be linked to the prescription of analgesics in NH residents with dementia, including physical health, activities of daily living, neuropsychiatric symptoms, prescription of psychotropic drugs, and demographics, which limited the risk of confounding. Furthermore, the study was conducted at admission to NH, with an annual follow-up for two years. This made it possible to assess whether length of stay in a NH had an impact on the prescription of analgesics or the relationship between pain severity and persistent prescription of analgesics.

Several limitations must also be mentioned. Firstly, the information regarding pain treatment was restricted. We did not have information about the systematic use of non-pharmacological treatment, which could preferably be included in a later observational study of pain treatment in NH residents with dementia. Others have stated that older adults with chronic pain may benefit from non-pharmacological treatment such as cognitive behavioral therapy, exercise, massage, music therapy, and reflexology [31, 60, 69, 70] for pain relief as well as for optimal treatment of diseases. The information regarding the prescription of analgesics (pharmacological treatment) in the present study could have been more detailed and included information about frequency and dosage of opioids and/or paracetamol prescribed, whether analgesics were taken, and information regarding analgesics as required, NSAIDs, polypharmacy, and the timepoint when the analgesics were prescribed at each assessment. As previously noted, information regarding effect and side effects of the analgesics prescribed is missing. Such information would contribute to a more thorough understanding of pain treatment in NH residents with dementia. Furthermore, participants not using analgesics at A_1_, A_2_, and/or A_3_ may have been prescribed these drugs between the assessment intervals. This information was unavailable for this study and may, therefore, limit the validity of the results.

Secondly, cancer and musculoskeletal disorders are diagnoses commonly found in older adults and related to pain [29], but information about diseases was missing in this study. Thus, associations between these diagnoses or other comorbidities related to pain and the outcome, and prescription and persistent prescription of analgesics could not be explored. However, the present study included information about general physical health in the analysis and found that poor general physical health was associated with the prescription of opioids when assessed simultaneously. Thus, we cannot guarantee that a reverse association is present, i.e., that opioids contribute to reduced physical health.

Thirdly, the definition of clinically relevant pain as baseline MOBID-2 ≥3, used in the initial analysis of this study to compare the prescription of analgesics at all three assessments and finding a persistent prescription of analgesics, may be considered a limitation. However, this was done to have a reference for the comparisons and was mandatory when the persistent prescription of analgesics between two consecutive assessments was assessed. Yet pain severity may change over time, and residents with clinically relevant pain at baseline may not experience clinically relevant pain at the follow-ups and vice versa. The degree to which these are the same residents who have clinically relevant pain at two or more consecutive assessments remains to be explored.

Lastly, a large number of NH residents (and thereby, potential participants) were not included for various reasons other than not having dementia and a life expectancy shorter than 6 weeks and furthermore some were excluded due to a significant amount of missing information, which may limit the study’s validity. Furthermore, data collection was performed in some but not all NHs in one of Norway’s counties. Thus, the sample is not necessarily representative for older adults with dementia admitted to NHs in Norway, and caution should be taken in generalizing the study results.

### Clinical implications

As demonstrated in this longitudinal, observational study of NH residents with dementia, pain is often present. Its treatment is demanding, and the prescription of opioids is particularly complex in this population [29, 31, 32]. All healthcare professionals involved in the care of such residents need to be aware of the challenges [71]. To improve quality of care and quality of life for NH residents with dementia, healthcare personnel must prioritize and improve the identification, monitoring, and treatment of pain [20] to reduce the prevalence and persistence of clinically relevant pain. Pain assessment at admission and regularly thereafter, with a valid and reliable pain assessment tool, should be performed routinely in NHs [72–75]. A systematic and reliable pain assessment is essential [27, 30]. For residents with dementia, a behavioral pain-assessment inventory like the MOBID-2 can be used to identify and manage pain [45, 76]. Routine pain assessments can reveal both undiagnosed and untreated pain and improve non-pharmacological and pharmacological pain treatment [18]. Systematic drug reviews [38] and interdisciplinary collaboration between nurses, physicians, and pharmacists are essential to effectively assess and treat pain and to evaluate the effect of pain treatment and identify potential side effects [31]. The application of criteria-based screening tools such as the STOPP/START criteria (Screening Tool of Older Person’s Prescriptions/Screening Tools to Alert Doctors to Right Treatment) [77] and the Norwegian General Practice–Nursing Home criteria (NORGEP-NH) [78] may contribute to appropriate prescribing and deprescribing of analgesics to NH residents with dementia. Future studies could explore innovative digital strategies for assessing pain intensity, pain treatment effects, and potential side effects in NH residents with dementia in order to address some of the clinical challenges pointed out.

## Conclusion

The prevalence and persistent prescription of analgesics were high among Norwegian NH residents with dementia. Paracetamol was most frequently prescribed at all assessments. The odds of prescribing opioids at follow-up were high if these were prescribed at baseline. More than a third of the studied residents had clinically relevant pain at NH admission. In adjusted analyses, higher pain intensity and poor physical health were associated with prescription and persistent prescription of analgesics. Interdisciplinary collaboration, routine assessment of pain at admission and regularly thereafter, and systematic drug reviews are essential to adequately assess and treat pain in NH residents with dementia.
